# Meta-Heuristic Algorithm-Tuned Neural Network for Breast Cancer Diagnosis Using Ultrasound Images

**DOI:** 10.3389/fonc.2022.834028

**Published:** 2022-06-13

**Authors:** Ahila A, Poongodi M, Sami Bourouis, Shahab S. Band, Amir Mosavi, Shweta Agrawal, Mounir Hamdi

**Affiliations:** ^1^ Department of Electronics and Communication Engineering, Sethu Institute of Technology, Kariapatti, India; ^2^ College of Science and Engineering, Hamad Bin Khalifa University, Qatar Foundation, Doha, Qatar; ^3^ Department of Information Technology, College of Computers and Information Technology, Taif University, Taif, Saudi Arabia; ^4^ Future Technology Research Center, College of Future, National Yunlin University of Science and Technology, Douliou, Taiwan; ^5^ John von Neumann Faculty of Informatics, Obuda University, Budapest, Hungary; ^6^ IAC, SAGE University, Indore, India

**Keywords:** breast cancer detection, computer-aided diagnosis, supervised learning, texture features, ultrasound imaging, wavelet neural network

## Abstract

Breast cancer is the most menacing cancer among all types of cancer in women around the globe. Early diagnosis is the only way to increase the treatment options which then decreases the death rate and increases the chance of survival in patients. However, it is a challenging task to differentiate abnormal breast tissues from normal tissues because of their structure and unclear boundaries. Therefore, early and accurate diagnosis and classification of breast lesions into malignant or benign lesions is an active domain of research. Over the decade, numerous artificial neural network (ANN)-based techniques were adopted in order to diagnose and classify breast cancer due to the unique characteristics of learning key features from complex data *via* a training process. However, these schemes have limitations like slow convergence and longer training time. To address the above mentioned issues, this paper employs a meta-heuristic algorithm for tuning the parameters of the neural network. The main novelty of this work is the computer-aided diagnosis scheme for detecting abnormalities in breast ultrasound images by integrating a wavelet neural network (WNN) and the grey wolf optimization (GWO) algorithm. Here, breast ultrasound (US) images are preprocessed with a sigmoid filter followed by interference-based despeckling and then by anisotropic diffusion. The automatic segmentation algorithm is adopted to extract the region of interest, and subsequently morphological and texture features are computed. Finally, the GWO-tuned WNN is exploited to accomplish the classification task. The classification performance of the proposed scheme is validated on 346 ultrasound images. Efficiency of the proposed methodology is evaluated by computing the confusion matrix and receiver operating characteristic (ROC) curve. Numerical analysis revealed that the proposed work can yield higher classification accuracy when compared to the prevailing methods and thereby proves its potential in effective breast tumor detection and classification. The proposed GWO-WNN method (98%) gives better accuracy than other methods like SOM-SVM (87.5), LOFA-SVM (93.62%), MBA-RF (96.85%), and BAS-BPNN (96.3%)

## 1 Introduction

Breast cancer is one among the most prevailing cancers diagnosed in women across the globe. It can occur in both men and women, but is more prevalent in women. According to the American Cancer Society (ACS) and World Health Organization (WHO), around 1.7 million people are diagnosed with breast cancer every year (1). Breast cancer can be treated and restored if it is detected at an early stage. Therefore, early detection of breast cancer is of prime importance for increasing the survival rate of patients (2). Screening is the best way of reducing mortality rate from breast cancer. Medical images are crucial sources of useful information. Several imaging techniques including mammography, ultrasound (US) imaging, and elastography are being widely used for diagnosing breast cancer (3, 4). Mammography is the most popular imaging technique for detecting cancer. However, it has some limitations like low sensitivity over ionizing radiations and is not suitable for dense breasts (5–8). Therefore, US or sonography is a valuable tool over mammography owing to its speed, inexpensiveness, and robustness (9), thereby it is safe and well tolerated by patients. Furthermore, US does not use any harmful radiation and has the ability to detect and differentiate benign from malignant tumors. Sonogram images are visualized, analyzed, and diagnosed manually by the physician or radiologist, but these are relatively tedious and time-consuming processes (10). In addition to this, manual analysis relies upon physician experience. Even experienced physicians may follow different diagnosis methods for manual analysis and have a sensitivity of 84% and specificity of 91% in breast cancer diagnosis. Therefore, there is a requirement to propose an efficient computer-aided diagnosis (CAD) system for US image analysis to enhance the diagnosis accuracy and help physicians make accurate timely decisions. An automated CAD system can help the physician to analyze the US image precisely and emphasize the suspected areas that need further investigation. Numerous methods for breast cancer diagnosis and developing sophisticated methods for enhancing the diagnosis accuracy have been carried out in the past years (11). A CAD involves five major phases: preprocessing, segmentation, feature extraction, feature selection, and classification (12). formulated a hybrid method for breast cancer diagnosis by integrating particle swarm optimization (PSO) and support vector machine (SVM) algorithms (13, 14).

PSO is mainly adopted for feature selection. The multiple instances learning-based breast cancer classification method is investigated by (15). This method is based on extracting the features from enhanced images and adopting SVM for segmentation (16). A self-organizing map (SOM) is used for converting the instance space to concept space. Finally, SVM is used for diagnosing breast cancer (17). proposed a classification approach for breast cancer diagnosis. Here, the US images are preprocessed with histogram equalization and the gamma correction method. Subsequently, texture features are extracted and classified as benign or malignant with the SVM classifier (18). reduced the speckle noise from breast US images by using an enhanced oriented despeckling anisotropic diffusion bilateral filter (19, 20). developed a method for increasing the classification accuracy. Here, the input images are preprocessed and segmented using the watershed algorithm. Morphological and texture features are obtained and fed as input to the classifier (21). employed an ANN for formulating the breast cancer diagnosis system. They computed feature scores based on breast imaging reporting and data system (BIRDS) criteria and then they used the biclustering method by (22, 23) to generate the hidden features. A hybrid model based on the fruit fly optimization algorithm (FLA) and SVM was assessed by (24). They employed FLA for tuning SVM parameters (25). The proposed particle swarm optimization, non-domination sorting with classifier technique model, and Bayes’ theorem help to select the most significant parameters and achieve optimum accuracy in breast cancer prediction (26). Jain et al. (2021) (27) proposes ensemble learning methods while making predictions. Bayes, K-nearest neighbors, AdaBoost, Gradient boosting, XGBoost, Random forest, ensembles, and neural networks are used for analyzing and predicting the dataset. It gives 87.248% and 87.7934% validation accuracy for predicting SARS79 CoV and SARS-CoV-2 virus respectively. The proposed algorithm produced the most accurate results, with 0.919 AUC and 87.248 percent validation accuracy for predicting SARS-CoV and 0.923 AUC and 87.7934 percent validation accuracy for predicting SARS-CoV-2 virus. Gupta et al. (2021) (28) proposed multimodal textual data consists of different emotions have been handpicked through the real time tweets related to COVID-19.After evaluating the most frequent word,modal vector has been created by using term frequency-inverse document frequency. Results showed that FLA-SVM achieved better classification accuracy. However, most of researchers have used machine learning models to perform classification tasks. Though machine learning is a powerful tool for image classification, it has some limitations like overfitting, increased learning time, and the fact that developing an optimal network structure is a tedious task and requires more time. Furthermore, optimizing parameters of the network is a difficult task. Parameter optimization is usually accomplished by experimentation. The major intention of this research is to propose a CAD system to diagnose breast cancer in an automated manner so as to overcome the limitations of the aforementioned existing techniques given in the literature. The major contributions of this work are as follows:

(1) To develop an automated CAD system by adopting a meta-heuristic algorithm and machine learning (2), to optimize the wavelet neural network with the grey wolf optimization algorithm to improve the classification accuracy in US breast cancer diagnosis, and (3) to compare the obtained outcomes with prevailing methods so as to project the preeminence of the proposed CAD system. The paper is structured as follows. Section 2 demonstrates the proposed CAD system for breast cancer. Section 3 provides experimental results and comparison with the existing methods. Section 4 concludes the paper.

## 2 Proposed Cad System

**Figure 1:**
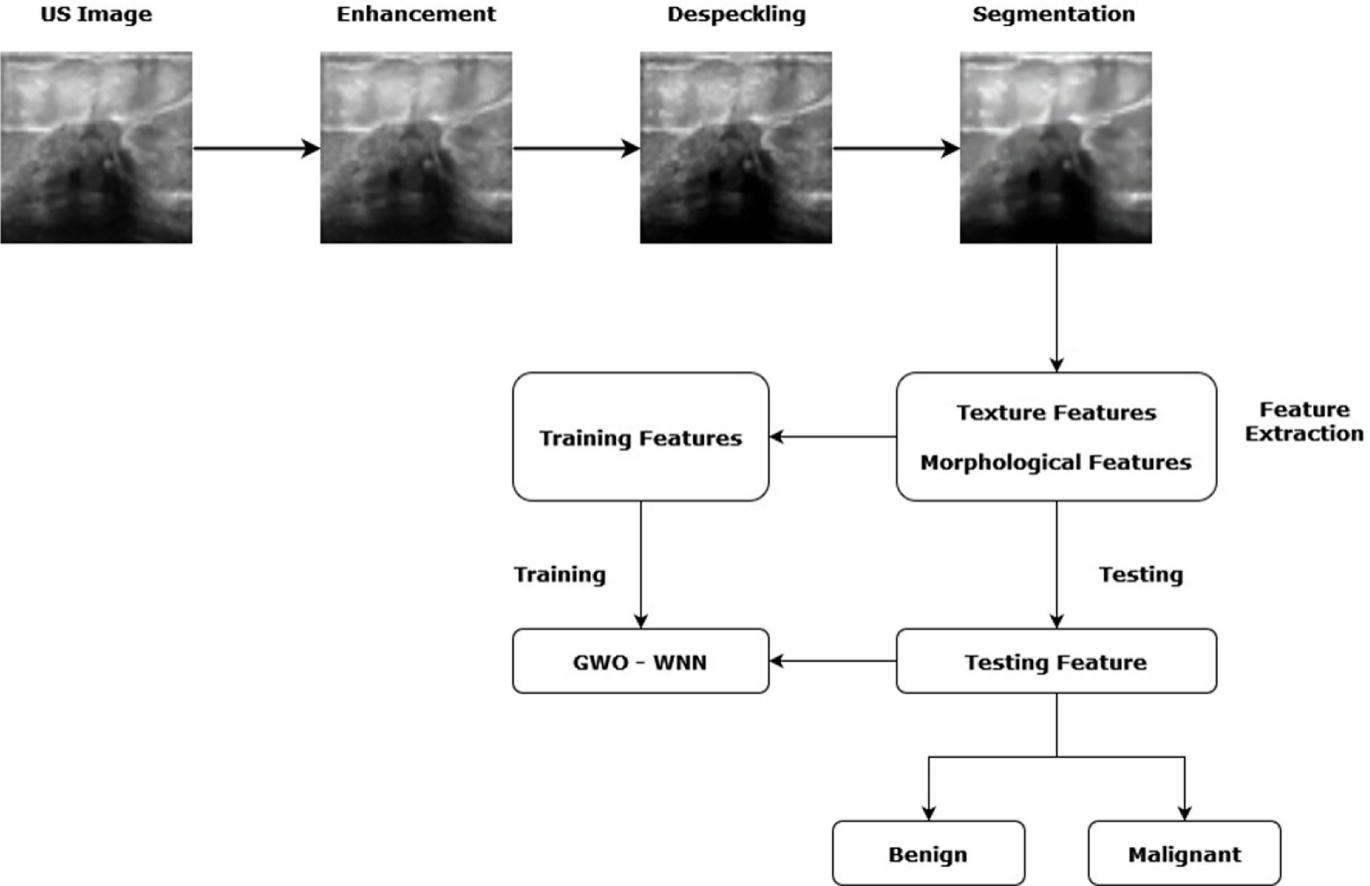
Articulation of the Proposed CAD system.

Research studies proved that ANN is an effective tool for breast cancer identification. Many researchers have employed ANN for developing a CAD system and showed comparatively better results. However, determining the optimal topology of ANN is a very difficult task. The number of hidden layers, hidden neurons, weights, and bias is usually determined by the trial-and-error method. This is a time-consuming process. To deal with this issue, bio-inspired algorithms like the genetic algorithm (GA), particle swarm optimization, and the fractional lion algorithm have been used for optimizing ANN parameters. The basic articulation of the suggested CAD system has been depicted in **Figure 1**.

### 2.1 US Image Preprocessing

Preprocessing is a crucial process in medical image processing. The aim of preprocessing breast US images is to improve the image quality and to remove unwanted information. **Table 1** shows the symbol’s nomenclature. In this research work, preprocessing involves two processes: enhancement and despeckling. A sigmoid filter is applied to breast US images to improve the quality. Let an input breast US image be assumed as *H*. Mathematically, the sigmoid filter can be expressed as Equation 1:


(1) $${\text{H}}_{\text{enh}}=\left({\text{H}}_{\text{mx}}-{\text{H}}_{\text{mn}}\right)\times \frac{1}{1+e^{\left[\frac{A-a}{Y}\right]}}+{\text{H}}_{\text{mn}}$$

**Figure 2:**
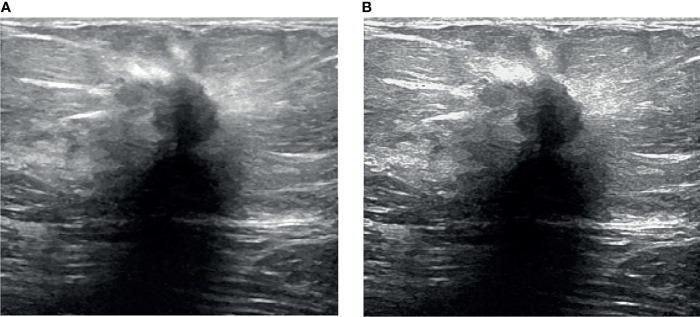
Processing results. **(A)** Ultrasound image and **(B)** enhanced ultrasound image.

Where, *H* is an input image, H*_enh_* is an enhanced image, mn is the minimum gray value, mx is the maximum value, and α and γ represent the center and width, respectively. In this paper, *lpha* = 4 and γ = 7 is used. **Figure 2** shows the processed images after enhancement.

**Table 1:** Nomenclature.

S.No	Symbol	Description
1	X	Input network
2	Y	Output network
3	H	Input breast US image
4	mn	Minimum gray value of the image
5	mx	Maximum gray value of the image
6	W	Weights between the layers
7	Ψ	Wavelet activation function
8	*α*	Center of the image
9	*γ*	Width of the image
10	*i*	Wavelet number
11	j	Wavelet level
12	k	Translation parameter
13	F	No. of features in the network
14	y	No. of classes in the network
15	Z	No. of hidden layers in the network
16	b	Bias
17	D	Modified distance vectors
18	C,E	GWO parameters

After enhancement, interference-based despeckling followed by anisotropic diffusion (IDAD) is applied to the enhanced image to remove speckle noise. The bright and dark regions of the US image represent constructive and destructive interference, respectively. The IDAD filter will suppress only destructive interference without losing any image information. The function of IDAD can be divided into two processes: median filter and interference suppression. A median filter with a kernel size of *n* ∊ *n* is applied to the enhanced image. Image pixels are filtered with an *n* ∊ *n* kernel around each pixel, replacing the central pixel value with the central value of all the pixels present in the kernel, and are mathematically represented in Equation 2.


(2) $$H_{\text{med }}\left(X,Y\right)=\underset{i,j\in k_{n,n}}{\text{med}}\left[k_{n,n}\left(i,j\right)⊗H_{\text{enh}}\left[X+i,Y+j\right]\right]$$

To prevent over-filtering, the kernel size was set to 3. Destructive interference suppression is expressed in Equation 3.


(3) $$H_{c}\left(X,Y\right)=\text{max }\left(H_{\text{enh}}\left(X,Y\right),H_{\text{med}}\left(X,Y\right)\right)$$

**Figure 3:**
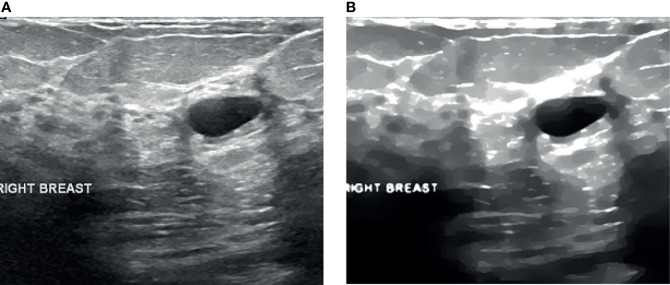
Despeckling results. **(A)** Raw US image and **(B)** filtered image.

Where, A*_c_* denotes the constructive interference image. Constructive interference suppression done by applying the median filter is applied to H*_c_* to remove the remaining extreme single pixels. **Figure 3** shows the breast US image after despeckling. It was clearly observed that the IDAD filter preserves the edges and boundaries of the image.

### 2.2 Segmentation

Image segmentation plays a significant role in breast cancer diagnosis. It is mainly employed to isolate the lesion region from its background. In recent years, clustering methods such as fuzzy C-means (FCM) and k-means algorithms were adopted for image segmentation. The FCM algorithm can separate lesions precisely. This algorithm is sensitive to noise and other artifacts. On the other hand, the k-means algorithm is faster than FCM but it has some limitations like incomplete detection of malignant lesions. This research presents an automatic segmentation method by combining the FCM and k-means algorithms in order to obtain accurate segmentation. The despeckled image is used as input in the segmentation process. The proposed segmentation method starts with initialization of the number of clusters, M, maximum iterations, and the termination parameter. The cluster centers can be mathematically expressed in the following Equations 4 and 5:


(4) $$\text{Initial}\;\text{mean}\;=\frac{\left(1:M\right)*n}{M+1}$$

and,


(5) n=max (US image )+1

**Figure 4:**
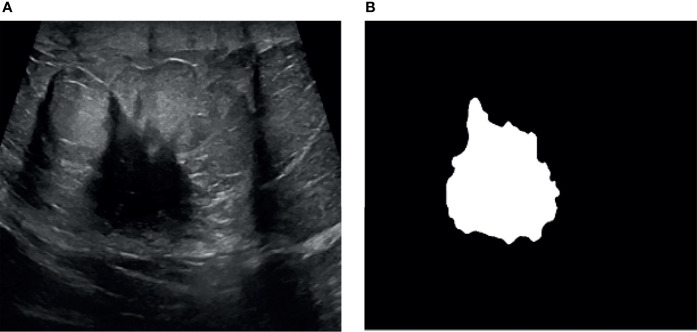
ROI segmentation. **(A)** Ultrasound image and **(B)** segmented image.

We assign each pixel to the nearest cluster center according to the minimum distance by examining the distance between the cluster centers and pixel value, and then recalculate the new cluster centers. This process repeats until the termination criterion is met. Both soft and hard clustering are used in the cluster formation. Hard clustering assigns each point based on the nearest cluster, in contrast, soft clustering assigns every point based on the degree of membership value. The clustered image is converted into a binary image with the thresholding method. Image binarization is one of the essential processes in image segmentation. The output of the binarization is the segmented image with a dark background and white foreground (lesion region). Active contour methods have been employed for segmentation and boundary tracking. In this research, active contour by the level set method is applied to track the boundary of the segmented lesions. The active contour by the level set method starts with initial boundary shapes represented as contours and is iteratively changed by executing shrinkage or expansion based on the constraints. The main advantage of active contour by level set is dividing the image into sub-images with continuous boundaries. A sample outcome of the segmentation process is shown in **Figure 4**.

### 2.3 Feature Extraction and Selection

**Figure 5:**
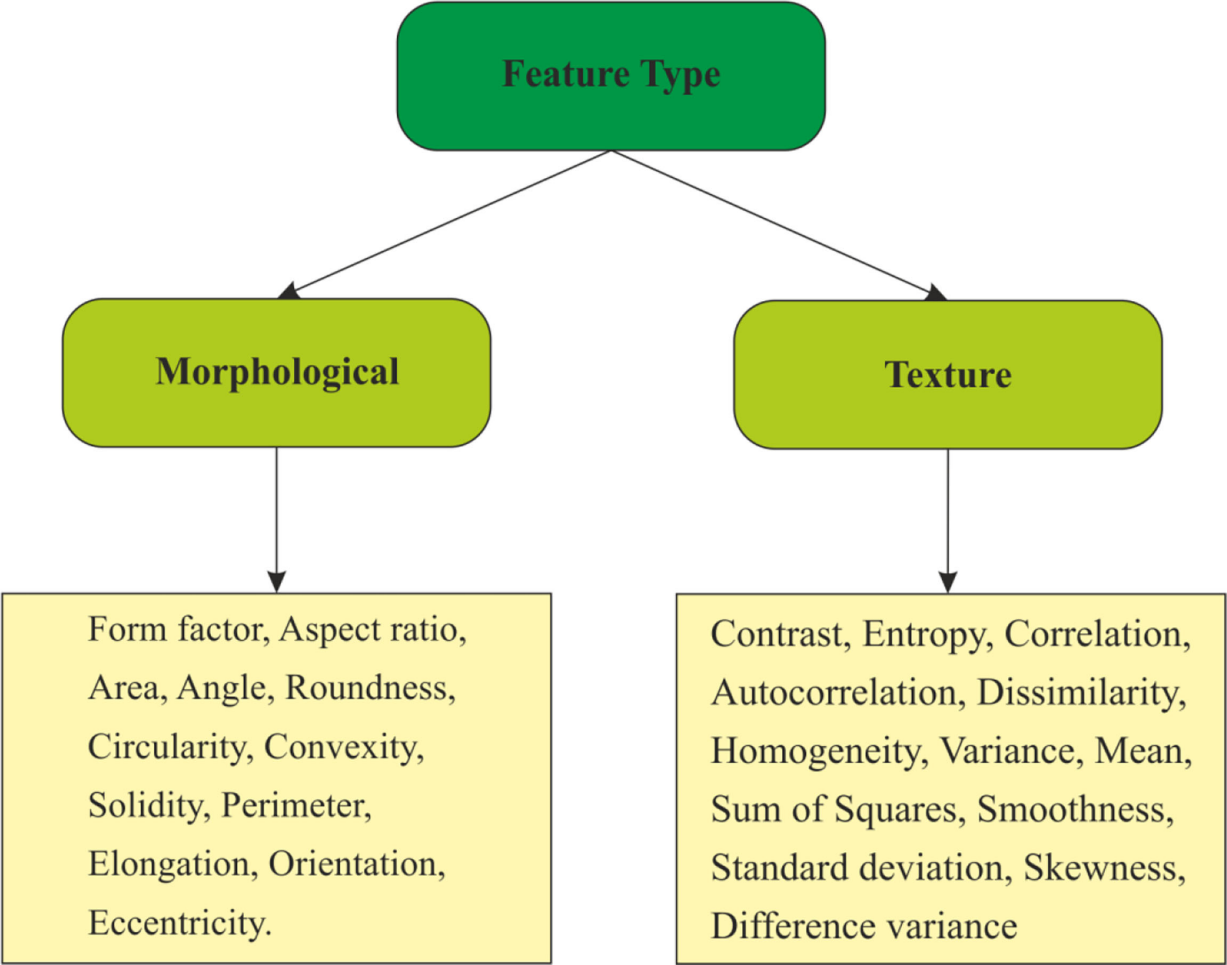
Selected features for this proposed scheme.

The crucial steps in breast cancer classification are feature extraction and feature selection. It is well known that benign and malignant lesions differ in both shape and pixel values. It is important to select the features related to shape and boundary of the lesion. Morphological features depict the characteristics such as shape and border of the lesion. Benign tumor margins are smoother, round or oval-shaped, and regular, whereas, malignant margins are angular, irregular shaped, and edged. It is also important to consider texture features for breast cancer classification since there are differences in boundary and internal echoes of malignant and benign lesions in US images. Therefore, the proposed CAD system employs two features to conduct differentiation amid benign and malignant lesions. Various texture and morphological features that were selected for conducing this experiment are provided in **Figure 5**. The obtained morphological and texture features are combined in order to obtain a single feature set. Principal component analysis (PCA) has been adopted to reduce the size of the combined features so as to speed up the training and testing processes, and also to improve the classification rate.

### 2.4 Classification

Classification has been identified to be applicable in different domains including medical image classification. The classification task largely relies on how efficiently the discriminant features and classifiers were selected. In recent years, numerous computational intelligence (CI) techniques such as the feed forward neural network, SVM, and wavelet neural network (WNN) have been used for image classification due to their ability to learn and find the relation between input and output variables *via* a training process. However, the feed forward neural network is largely popular and the most commonly used to diagnose medical images. Wavelet is a mathematical tool used to analyze a signal or an image in different resolutions. It offers the potential of obtaining spatial and frequency domain features of a signal or an image. This paper develops a classifier by combining a neural network and the wavelet theory WNN to obtain a higher classification rate when compared to its peers. The peculiar advantages in using WNN when compared to multilayer perceptron (MLP) and SVM include the fact that WNN can handle non-linear, noisy, and periodic signals. Thus, it works well in determining the abnormalities in breast US images. Moreover, WNNs are robust and require only smaller training samples for tuning the parameters when compared with MLP and SVM. Also, WNNs offer faster convergence when compared with MLP and SVM. Hence, this proposed method employs WNN owing to these aforementioned enhanced features.

**Figure 6:**
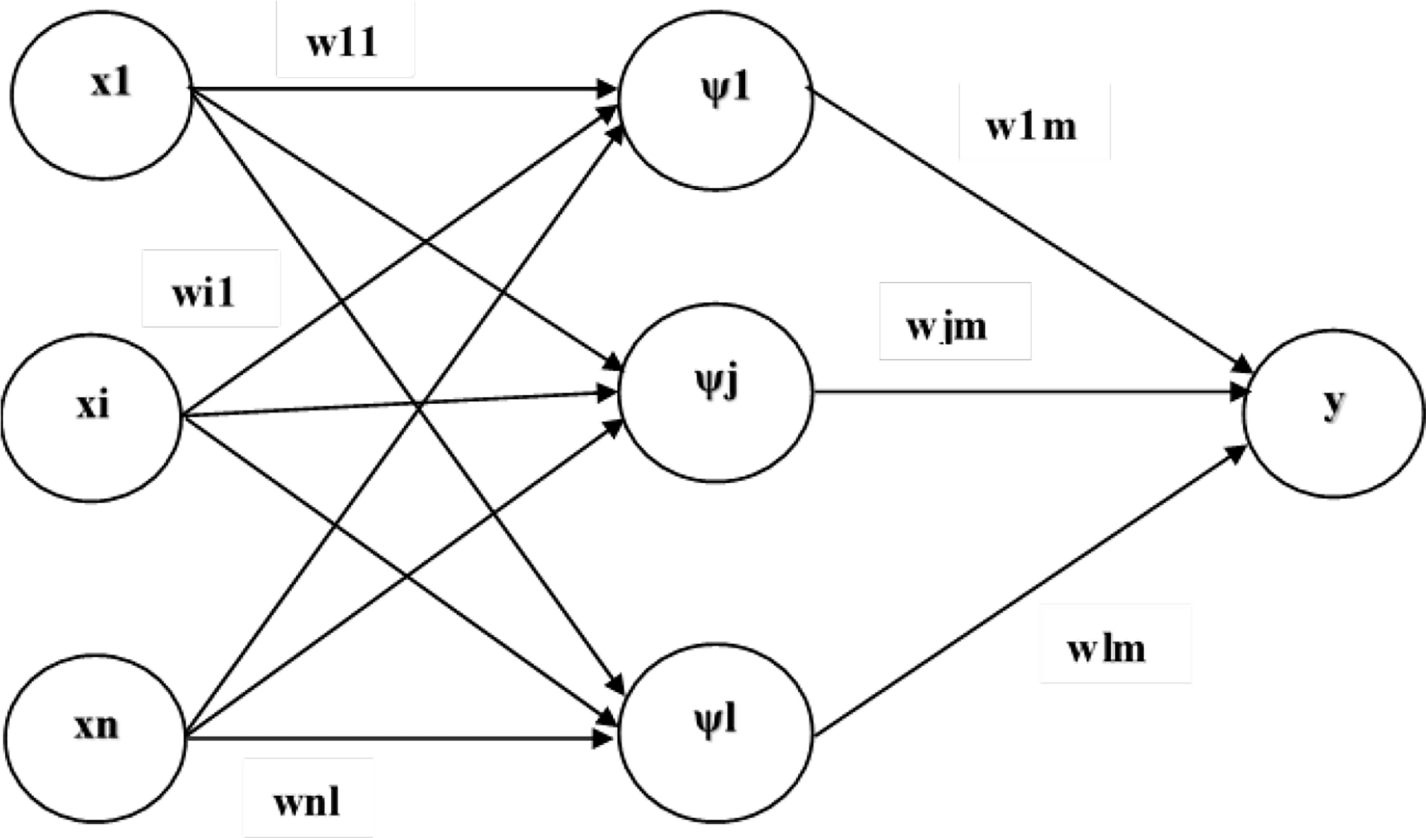
Structure of WNN.

**Figure 6** shows the general structure of WNN. A typical WNN has three layers which are the input, hidden, and output layers. The input layer is responsible for receiving input signals or feature vectors from the source. The hidden layer has hidden neurons similar to neurons in standard neural networks (NNs). In standard NNs, the sigmoidal activation function is utilized at the hidden layer. Wavelets are subsequently incorporated as the activation function in the hidden layer of WNNs. Finally, the output layer corresponds to the linear combination of the wavelet basis function. The error between predicted values and expected values is calculated by cost function, and also represents itself in the format of a single real number. Based on the nature of problems, the cost function could be framed in various ways. There is no standard rule for selecting the mother wavelet function as the activation function.

In general, there are numerous categories or families of wavelets such as Daubechies, Symlets, Coiflets, biorthogonal, Haar wavelets, etc. In this paper, the Haar wavelet is employed owing to its characteristics toward simple structure and increased memory efficiency. The Levenbergmarquart (LM) algorithm is employed for minimizing the error and also to update the weights of WNN. It is noted that psi signifies the wavelet activation function. In a standard neural network, the tan sigmoidal activation function is used at the hidden layer. But, in the case of a wavelet neural network, wavelets are employed as the activation function, thus, psi corresponds to the mother wavelet.

Let the input signal be represented as X={x_1_, x_2_, x_3_,…,x*_n_*} and the output signal be signified as Y={1, 2, 3, …, m}, with w*_ij_* and w*_jk_* corresponding to the weights between the layers. Ψ is the wavelet activation function. The weighted summation of the wavelet basis function at the hidden layers is closely related to the output layer. The output of the network with respect to the corresponding Haar wavelet function can be represented in the following Equations 6 and 7:


(6) $$\text{Yp}=\sum_{\text{j}=1}^{\text{I}}{\text{W}}_{\text{jk}}\text{Ψab}\left[\sum_{i=1}^{\text{n}}{\text{X}}_{\text{ij}}{\text{W}}_{\text{ij}}+{\text{bi}}_{\text{j}}\right]$$

(7) $${\text{Ψ}}_{ab}\left(net_{j}\right)=\text{Ψ}\left[\frac{net_{j}-b_{j}}{a_{j}}\right]$$

Where a and b are the wavelet scale and translation parameter of the j*_th_* hidden neuron. Although WNN is a promising tool for pattern recognition, optimizing the parameters of WNN like learning rate, weight, number of hidden neurons, etc., are quite difficult. The features of the Haar wavelet function that was employed in this proposed method are described below.

Let us assume, m = 2, *k* = 0,1,1,3,… .*m* – 1,*j* = 0,1,2,3, and *i* = *m* + *k* + 1. Where i signifies the wavelet numbers, j corresponds to the wavelet levels, J signifies the highest resolution, and k corresponds to the translation parameter. The highest value of i is selected as i=2M and the lowest value is selected as i=2. The typical Haar wavelet families corresponding to *t* ∊ [*A*,*B*] shall be mathematically expressed in the following Equations 8 and 9,


(8) $$h_{i}\left(t\right)=-\begin{array}{ccc} 1 & \text{for} & t\in \left|\xi_{1}\left(i\right),\xi_{2}\left(i\right)\right|\\ 1 & \text{for} & t\in \left|\xi_{2}\left(i\right),\xi_{3}\left(i\right)\right|\\ 0 & \text{for} & \text{otherwise} \end{array}$$

Here, 

$\begin{array}{cc} \xi_{1}\left(\text{i}\right)=\text{A}+2\text{k}\mu \text{Δx},\xi_{2}\left(\text{i}\right)=\text{A}+\left(2k+1\right)\mu \text{Δx}, & \text{and }\xi_{3}\left(\text{i}\right)=\text{A}+\left(2k+1\right)\mu \text{Δx} \end{array}$


(9) $$\text{Also},\mu =\frac{M}{m}\;\&\text{Δ}x=\frac{B-A}{2M}$$

The scaling function h_1(t)_ shall be mathematically signified in Equation 10:


(10) $$h_{1}\left(t\right)=\{\begin{array}{c} 1\;\text{for}\;t\in \left[A,B\right]\\ 0\;\text{for}\;\text{otherwise}\; \end{array}$$

In general, the Haar wavelets are orthogonal with respect to each other. Hence, the orthogonality property shall be specified in Equation 11:


(11) $$\int_{\text{A}}^{\text{B}}h_{\text{i}}\left(\text{t}\right)\times {\text{h}}_{\text{s}}\left(\text{t}\right)=2^{\text{i}}\delta_{\text{ij}}=\{\begin{array}{cc} 2^{\text{j}} & \text{i}=\text{s}=2^{\text{j}}+\text{k}\\ 0 & \text{i}\neq \text{S} \end{array}$$

The orthogonality property of this Haar wavelet is mainly employed to enhance the efficacy of the present method. The basic function y(t) within the interval [A,B] shall be extended to the Haar wavelet and this wavelet extension shall be expressed in Equation 12:


(12) $$\text{y}\left(\text{t}\right)=\sum_{\text{i}=1}^{2\text{M}}{\text{a}}_{\text{i}}{\text{h}}_{\text{i}}\left(\text{t}\right){\text{ \&a}}_{\text{j}}=2^{\text{j}}\int_{\text{A}}^{\text{B}}\text{y}\left(\text{t}\right)\times {\text{h}}_{\text{i}}\left(\text{rmt}\right)\text{dt}$$

To address the aforementioned limitations, the GWO algorithm is employed for tuning the WNN parameters. GWO is a category of nature-inspired algorithms motivated by the hierarchical organization and hunting attitude of grey wolves. GWO was initially formulated by (29). All grey wolves like to live as a group, thereby this GWO optimization algorithm possesses a social dominant hierarchy.

In a GWO algorithm, α represents the leader wolf since his/her order should be obeyed by the group. Here, α is responsible for making the decision regarding hunting, sleeping place, and so on. The *β* wolf should respect the leader, but commands *δ* and ω. Delta wolves should respect α and *β* , but dominates ω. The ω wolves have to submit to *α*, *β*, and *δ*. It was observed that the GWO algorithm possesses good exploration and exploitation characteristics when compared to particle swarm optimization, the genetic algorithm, and the cat swarm optimization algorithms. The corresponding pseudo code for the GWO algorithm which was employed in this experiment is given in the following Equations 13-23.

Initialize search agents, *S* randomly, variables *v*, and maximum iteration**s,** I*_max_*, *a*, *C*, and *E*.


(13) $$\begin{array}{l} D^{U}=\left|E^{u}X^{u}p\left(t\right)-X^{U}\left(t\right)\right| \end{array}$$

(14) $$X^{u}\left(t+1\right)=X^{u}p\left(t\right)-C^{u}\cdot D^{u}$$

(15) $$C^{u}=2.a.r1-a$$

(16) $$E^{u}=2.r2$$

Evaluate the fitness values of each agent.

X_α_ – First prime agent,

X_β_ – Second prime agent ,

X_δ_ -Third prime agent


(17) $$D_{a}^{U}=E_{1}^{u}.X_{\alpha }^{u}-X^{u}$$

(18) $$D_{\beta }^{U}=E_{2}^{u}.X_{\beta }^{u}-X^{u}$$

(19) $$D_{\delta }^{U}=E_{1}^{u}.X_{\delta }^{u}-X^{u}$$

(20) $$X_{1}^{u}=X_{\alpha }^{u}-C_{1}^{u}\cdot \left(D_{\alpha }^{u}\right)$$

(21) $$X_{2}^{u}=X_{\beta }^{u}-C_{1}^{u}\cdot \left(D_{\beta }^{u}\right)$$

(22) $$X_{3}^{u}=X_{\delta }^{u}-C_{1}^{u}\cdot \left(D_{\gamma }^{u}\right)$$

While (*il_max_*)for *j* = 1:*S*

Update position


(23) $$X^{u}\left(t+1\right)=\frac{X_{1}^{u}+X_{2}^{u}+X_{3}^{u}}{3}$$

End for

Update *a*,*C*,*E*

Compute the quality of each search agent.

Update X_α_, X_β_, X_δ_

i=i+1

End while

return X_α_

In this proposed method, GWO-WNN is employed for developing WNN and is tuned with the best topology by tuning the weights, number of hidden layers and neurons, and respective learning rate. The algorithmic steps used to train and classify the breast US images are given in the following steps

**Step 1:** Read breast US image, *H*.
**Step 2:** Convert 3D image to 2D image.
**Step 3:** Enhance the quality of the image using Equation (24).
**Step 4:** Despeckle the enhanced image using Equation (26).
**Step 5:** Perform segmentation.
**Step 6:** Extract the features listed in **Figure 5**.
**Step 7:** Design a classifier.

F*i* represents the count of features, y*_o_* corresponds to the count of classes, and Z*_n_* represents the count of hidden layers. The GWO algorithm determines the count of hidden neurons in each hidden layer weight and learning rate. Let g be the transfer function and it can be expressed in the following Equations 24 and 25.


(24) Yp=g(Zj)

(25) $$Z_{j}={\text{Ψ}}_{ab}\sum_{j-1}^{n}X_{i}W_{ij}+b_{j}$$

$Z_{p}^{j}=\sum_{v}W_{v\cdot p}y_{v}^{j}+B_{p}$ Where, j represents the j*^th^* hidden neuron, y*_p_* is the output of the p*^th^* neuron, W*_i,j_* is the weight, and b denotes the bias.

In this research work, the hidden layer and output layer used the wavelet activation function and linear activation function accordingly. Mean square error (MSE) is utilized as the fitness function for training, MSE can be expressed in Equation 26:


(26) $$\begin{array}{l} \text{MSE}=\sum_{j\in t}\sum_{\text{p}=1}^{{\text{Y}}_{\theta }}{\left({\text{t}}_{\text{p}}^{\text{j}}-{\text{y}}_{\text{p}}^{\text{j}}\right)}^{2} \end{array}$$

**Step 8:** Optimize the parameter of WNN

Where ${\text{t}}_{p}^{j}$ is the labeled class and ${\text{y}}_{p}^{j}$ is the output obtained from the network.

## 3 Results And Discussion

### 3.1 US Image Dataset

**Figure 7:**
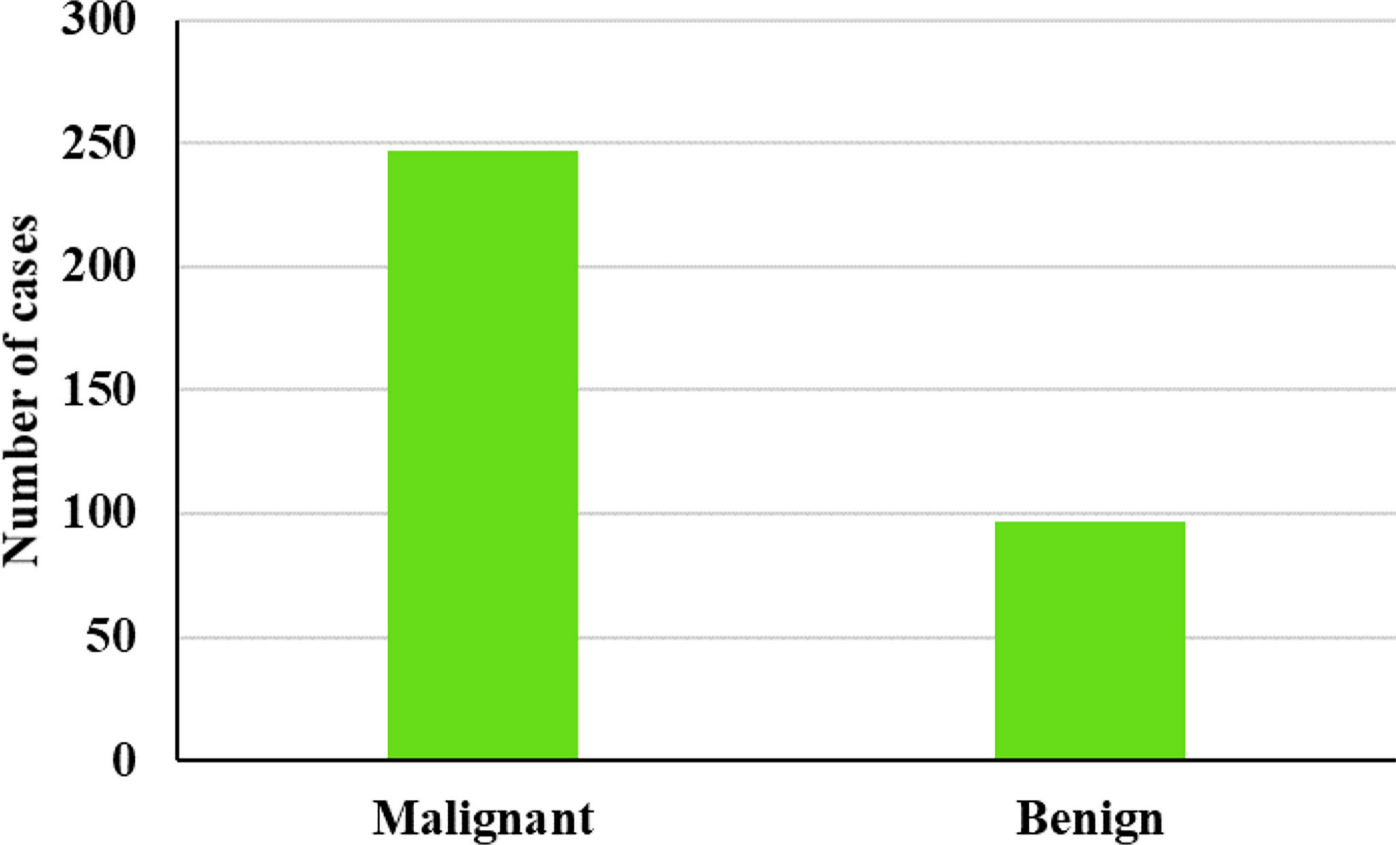
US image dataset.

Breast US images were acquired from 346 patients from the database given below (https://scholar.cu.edu.eg/q=afahmy/pages/dataset) (30) including 249 malignant and 97 benign samples employed for assessing the effectiveness of the proposed GWO-tuned WNN. The entire dataset was divided into two sets: 70% of the data were utilized for training and the rest of the 30% were used for testing the classification ability of the system. A 10-cross fold validation was considered to test the efficiency of the presented system. The number of benign and malignant cases used in this research work is graphically displayed in **Figure 7**. The proposed scheme was implemented using MATLAB so as to analyze the efficacy of the presented system when compared to its peers.

### 3.2 Evaluation Indices

This section describes the evaluation parameters that were employed to analyze the efficacy of the presented scheme. Accuracy of the classification, specificity, sensitivity, positive predictive value (PPV), negative predictive value (NPV), confusion matrix, and ROC were employed to analyze the classification performance.

### 3.3 Performance Evaluation

**Figure 8:**
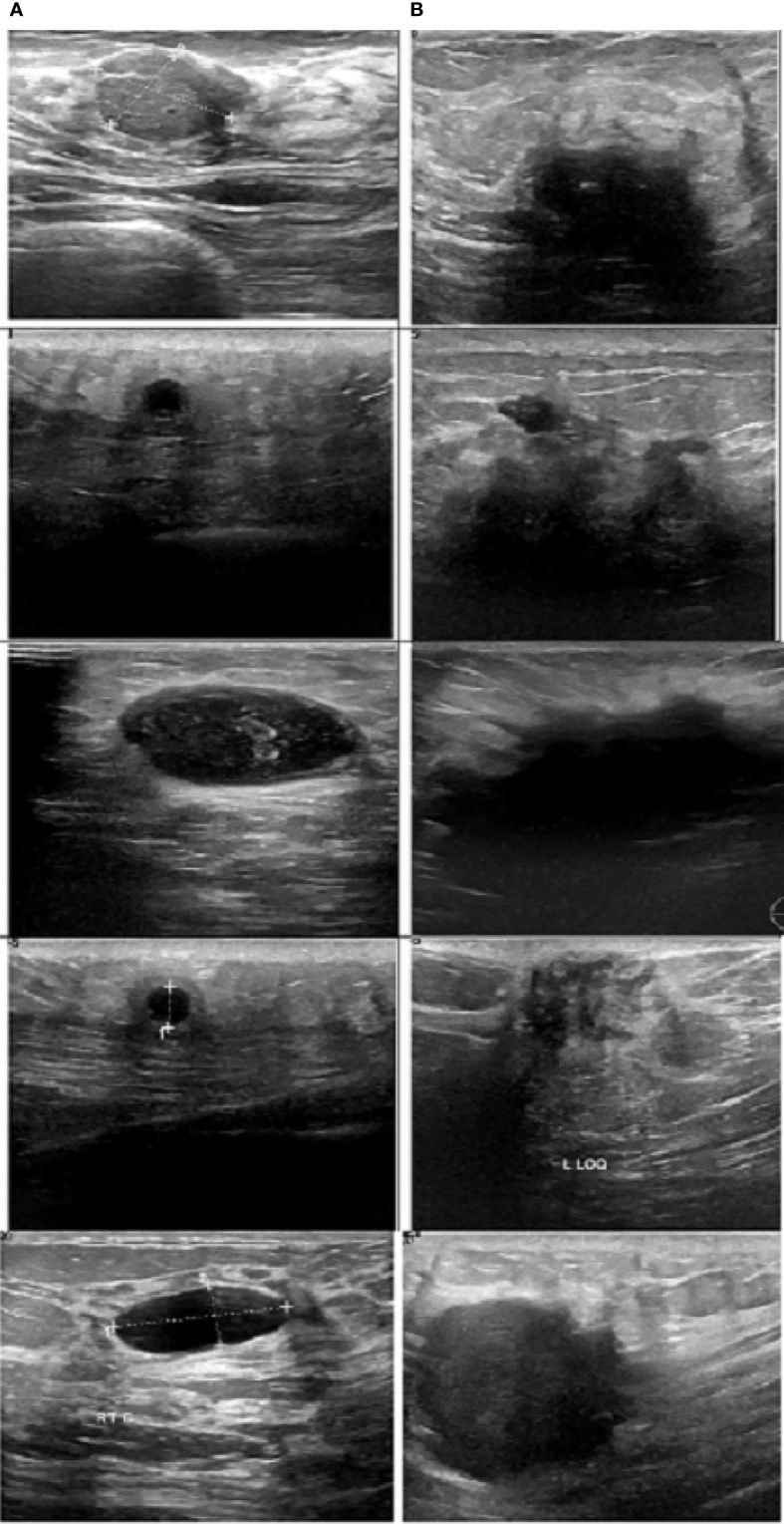
Sample images. **(A)** Benign and **(B)** malignant (30)

**Figure 9:**
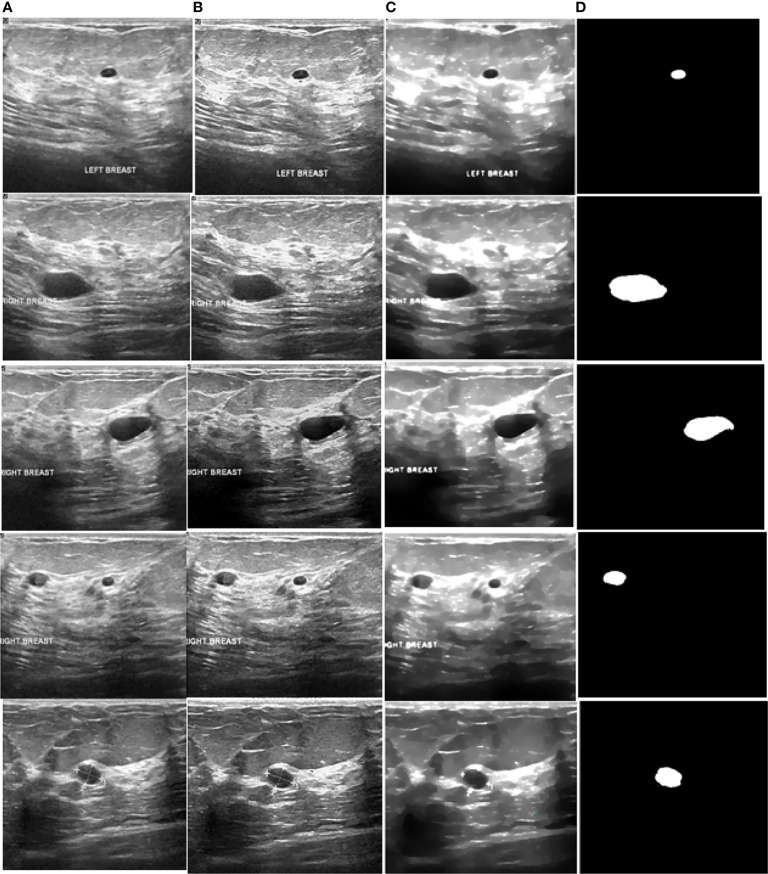
Processed benign images. **(A)** US image, **(B)** enhancement, **(C)** despeckling, and **(D)** Segmentation.

**Figure 10:**
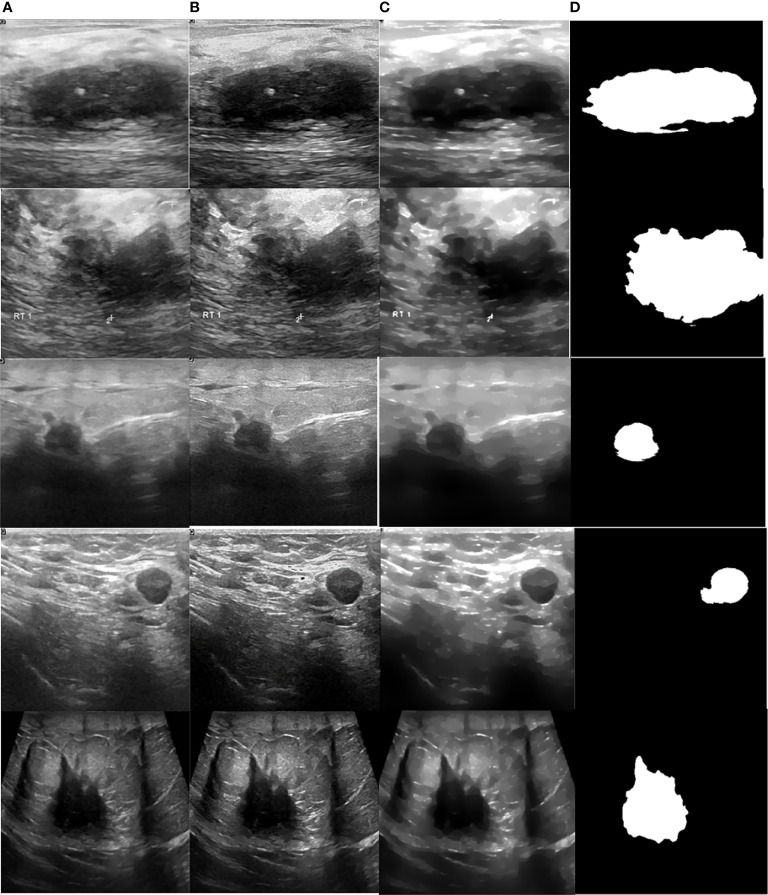
Processed malignant images. **(A)** US image, **(B)** enhancement, **(C)** despeckling, and **(D)** Segmentation.

**Figure 11:**
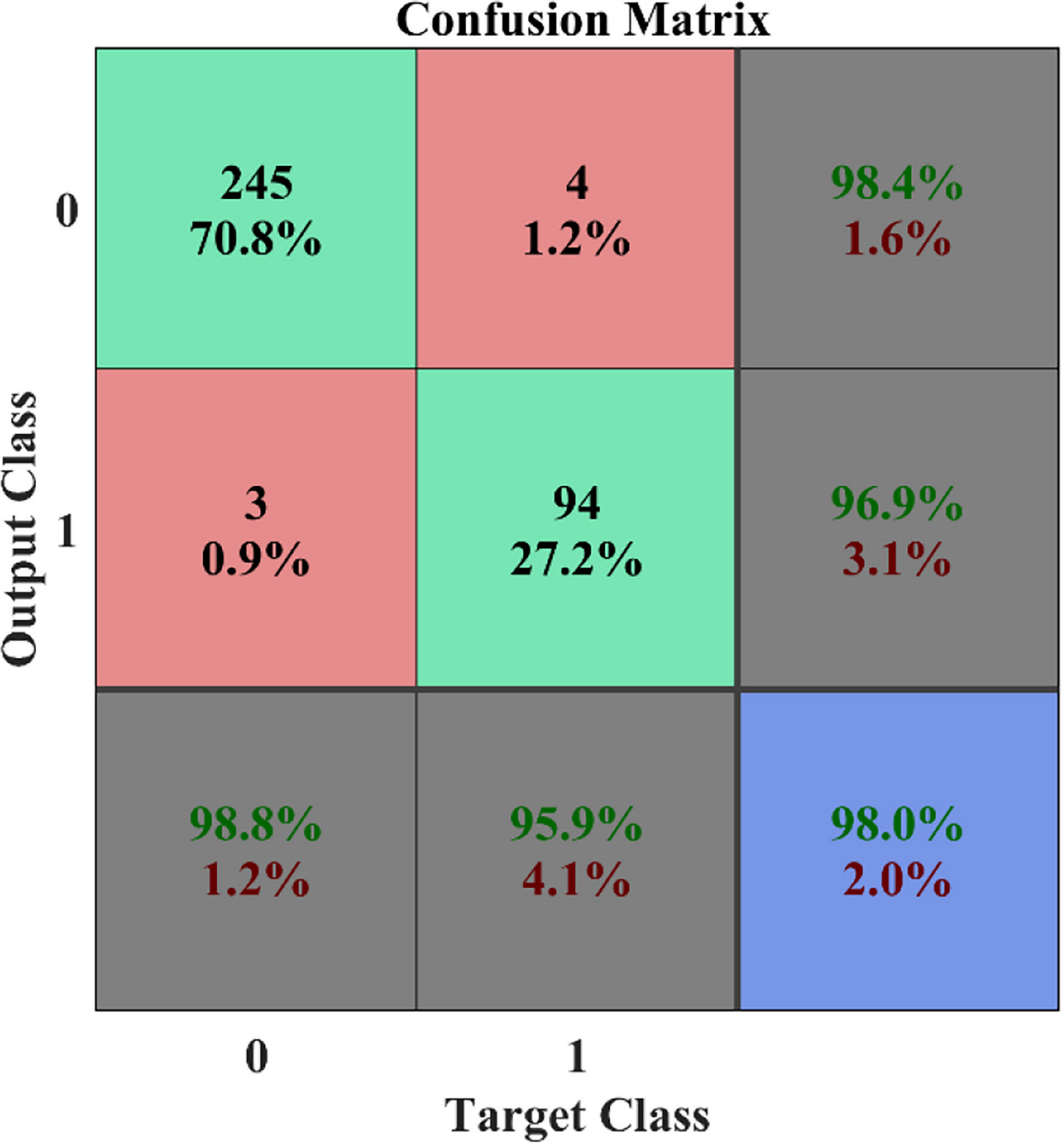
Confusion matrix.

**Figure 8** illustrates a variety of benign and malignant breast ultrasound pictures. The preprocessed 257 benign and malignant US pictures are shown in **Figures 9** and **10**, respectively. **Figure 11** depicts the confusion matrix to discriminate the cancer (malignant) from non-cancer (benign) breast lesions. In this research work, malignant tumors were defined as true positive and benign as true negative. From **Figure 11**, it can be observed that 245 out of 249 images were correctly diagnosed as malignant tumors. Likewise, 94 out of 97 images were correctly identified as benign. Consequently, true positive, false positive, false negative, and true negative values were obtained at 70.8%, 1.2, 0.9%, and 27.2%, respectively. The results exhibit superior classification accuracy of 98% with a sensitivity of 98.8%, specificity of 95.9%, PPV of 98.4%, and NPV of 96.9%.

This paper evaluated some widely used performance indicators such as the confusion matrix, receiver operating characteristic (ROC), classification accuracy, sensitivity, and specificity. Accuracy calculated the proportion between true positive and total data, and shall be mathematically represented in the following Equations 27, 28, and 29:


(27) $$\begin{array}{l} Accuracy=\frac{TP+TN}{TP+TN+FP+FN} \end{array}$$

(28) $$Specificity=\frac{TN}{TN+FP}$$

True positive is represented by TP, true negative is represented by TN, false negative is represented by FN, and false positive is represented by FP.


(29) $$Sensitivity=\frac{TP}{TP+FN}$$

### 3.4 Comparison With the State-of-the-Art Existing Methods

In this paper, a CAD model in breast cancer diagnosis was proposed and implemented. The efficiency of the presented CAD system was analyzed with the current methods: SOM-SVM (15), LFOA-SVM (24), MBA-RF (31) and BAS-BPNN (32) in terms of statistical metrics and ROC curve.

**Figure 12:**
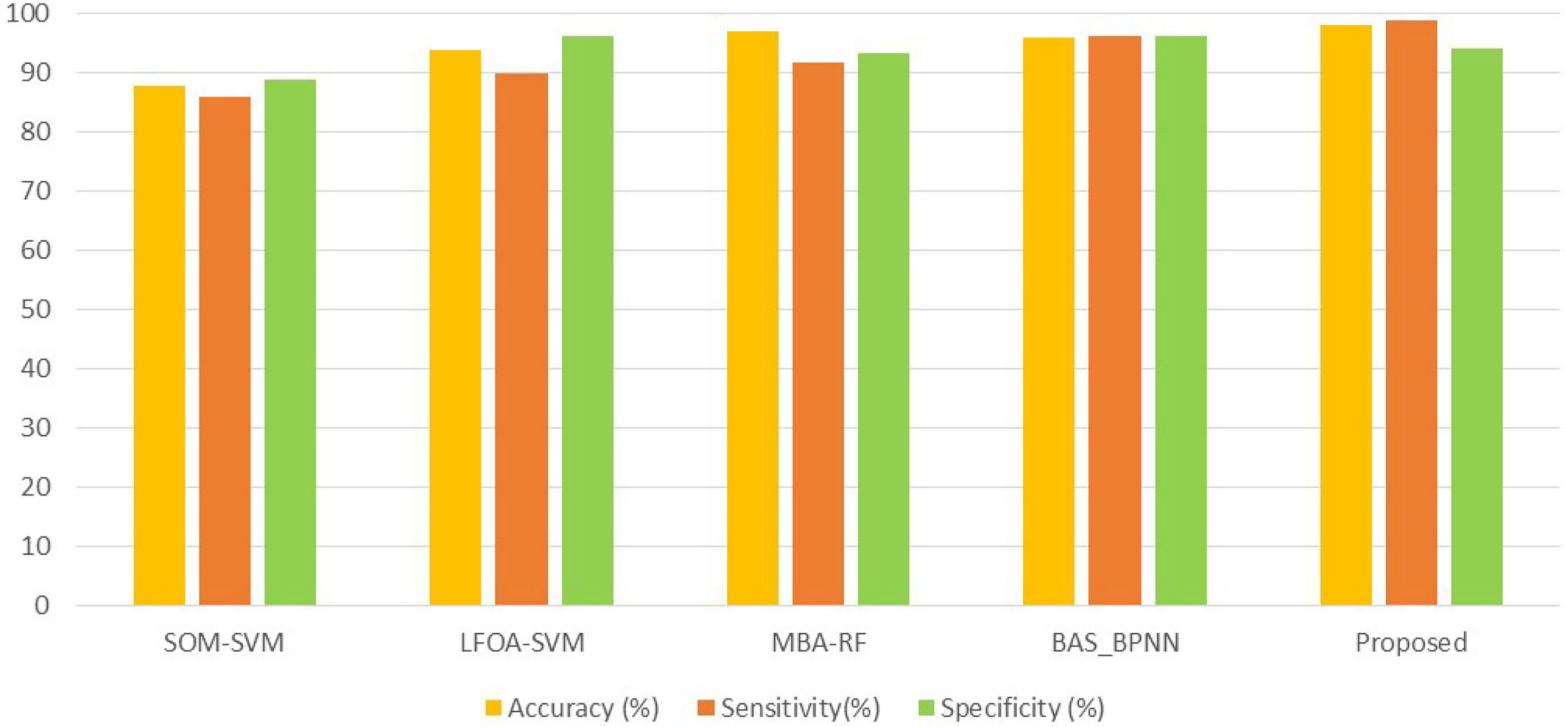
Performance comparison with existing methods.

**Figure 12** and **Table 2** display the classification performance of the proposed GWO-tuned WNN with the existing classifiers SOM-SVM, LFOA-SVM, MBA-RF, and BAS-BPNN (15). integrated SOM and SVM [SOM-SVM] for developing a breast cancer diagnosis system. Here, the input images were preprocessed and divided into ROIs. The segmented ROIs and subregions were considered as bags and instances of bags, respectively. Subsequently, SOM was used for converting the instance space to concept space. Finally, SVM was incorporated for classifying the breast lesions. The major limitation here is that the method uses only local features for classification. Global features are also equally important for breast cancer diagnosis, thereby this is considered as a main drawback in this approach.

**Table 2:** Classification performance of the proposed GWO-tuned WNN with existing classifiers.

Contributors	Data	Method	Accuracy(%)	Sensitivity(%)	Specificity(%)
(15)	US image	SOM-SVM	87.5	86.11	88.54
(24)	US images	LFO-ASVM	93.62	90	96.3
(31)	WDBC	MBA-RF	96.85	91.4	93.2
(32)	WDBC	BAS-BPNN	96.3	96.3	95.69
**Proposed** **method**	US images	GWO-WNN	98	98.8	95.9

(24) implemented a framework by using SVM for breast cancer diagnosis. Here, the fruit fly optimization algorithm (FLA) modified by the Levy flight optimization algorithm (LFOA) was adopted for tuning the SVM parameters. High level features were obtained, normalized, and applied as input to the LFOA-SVM. However, this framework resulted in higher computational overhead, which was considered to be a focal drawback in this approach.

(31) examined the features of the modified bat algorithm (MBA) in selecting the feature vectors for breast cancer diagnosis. MBA employs the random sampling method to select features from the input data. Selected features are ranked and further employed to train the random forest (RF) classifier. This MBA-RF method exhibited comparatively better outcomes than the correlation-based feature selection method.

(32) formulated a strategy by integrating bio-inspired algorithms (BAs) and the back propagation neural network (BAS-BPNN). Here, the input data were subjected to preprocessing, feature extraction/selection, and classification. Moreover, three BAs were typically employed for selecting the features. Each algorithm selected corresponding subsets of features. The selected features were combined and classified by using BPNN.

(33) formulated three subnetworks, of which the first one contained the ROI, the second one contained the filtered ROI, and the third one was spread to the whole image. The first subnetwork in the CNN model was formulated using a single block, whereas the second subnetwork was formulated by two blocks and the third network was formulated by three blocks.

For datasets acquired using different scanners with different imaging protocols (34), used an efficient method by transfer learning to fine-tune the classification task in order to improve the accuracy. The mean accuracy reached 0.9 by the CNN hence accuracy in testing increased from 0.47 to 0.78 using CNN.

(35) suggested that the mixture of –spatial spectral features boost the classification precision of the CNN model which needs considerably fewer training parameters comparable with the best known system.

(36) formulated a conventional binary SVM classifier which was trained with seven features such as color and texture attributes obtained from the Gaussian model super pixels of the images belonging to WCE.

Numerous methods have been developed to analyze the strength of meta-heuristic algorithms for selecting salient features to improve the classification rate. However, these methods have some shortcomings like increased training time, overfitting, and slow convergence. Very few researchers have used meta-heuristic algorithms for tuning the parameters by machine learning. Therefore, we used the GWO algorithm for tuning the parameters of WNN to reduce the computational overhead and training time. In the proposed system, 10-cross fold validation is considered to test the efficacy.

**Figure 13:**
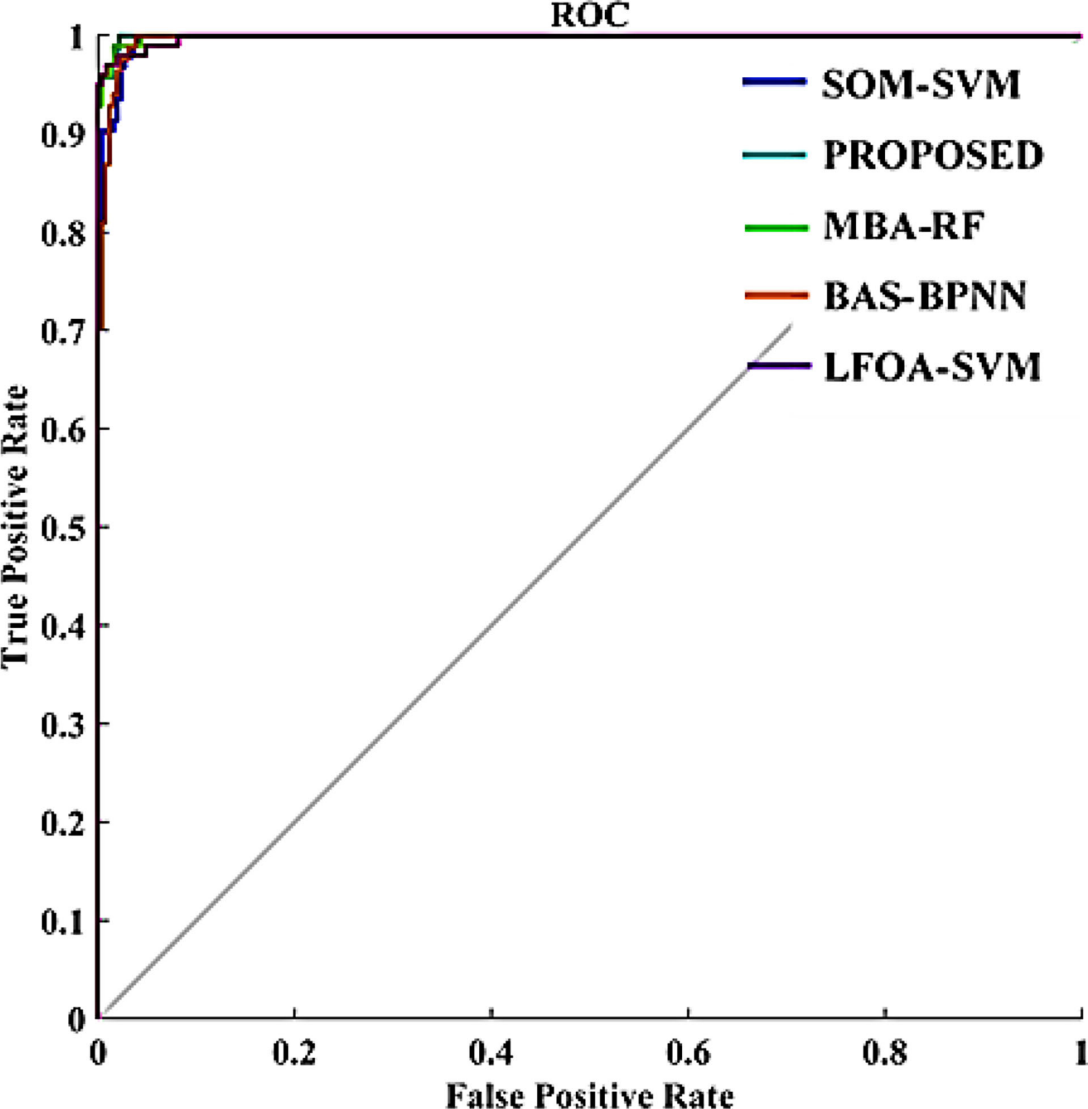
ROC curve comparison.

Almost all previous research works employed BAS for feature selection. But, in this paper, we employed the GWO algorithm for optimizing the WNN parameters. Here, optimization aids to reduce computational cost and improve classification accuracy. As depicted in **Figure 13**, it was observed that existing methods were 87.5% (SOM-SVM), 93.62% (LFOA-SVM), 96.85% (MBA -RF), and 96.3% (BAS-BPNN) accurate in terms of classification (15, 24, 31, 32).

However, the proposed CAD system attained a classification precision of 98% which outperforms the other existing models SOM-SVM, LFOA-SVM, MBA-RF, and BAS-BPNN. Comparison outcomes indicate that the performance of the proposed GWO-WNN is better than other classifiers. This is because of the important scenario in the presented method of integrating GWO and WNN, and tuning WNN parameters with the GWO algorithm. GWO has a natural leadership mechanism which makes it more effective than other approaches. **Figure 13** provides the comparative analysis in terms of the ROC curve of the proposed scheme with other classifiers. From **Figure 13**, it can be seen that the area under the curve of the presented CAD system is greater than that of the state-of-the-art methods like SOM-SVM, LOFA-SVM, MBA-RF, and BAS-BPNN (0.99, 0.91, 0.93, 0.92, and 0.95). Therefore, this particular result highlights the effectiveness of the GWO-tuned WNN in US image classification. As a result, classification accuracy was improved with the proposed method.

## 4 Conclusion

Ultrasound imaging techniques are an important diagnostic tool for breast cancer diagnosis. The CAD model can assist physicians in generating more precise decisions. This paper proposed a CAD model for breast cancer diagnosis by the integration of a wavelet neural network and the grey wolf optimization algorithm. In this system, a sigmoid filter was used to increase the contrast of the images and IDAD to remove the speckle noise. An ROI was obtained from the preprocessed image, and subsequently the morphological and texture features were extracted and combined. The dimensions of the obtained features were reduced with PCA. Finally, GWO-WNN was executed to perform the classification task. Conventional WNN requires increased time for training and optimizing the parameters. This proposed scheme employed GWO for tuning the WNN parameters which eventually reduced the computation and training times. The simulation results clearly showed that the suggested CAD model system attained desirable classification accuracy when compared to its peers with respect to the classification accuracy. The leading advantages of the proposed GWO-WNN method include increased robustness, smaller training data, and faster convergence. Ultimately, since the GWO algorithm has a natural leadership mechanism, it makes it superior to other bio-inspired algorithms. Most of the former methods have employed a meta-heuristic algorithm for feature selection. But, the proposed CAD system used the meta-heuristic algorithm to tune the WNN parameters which helped to reduce the computational cost and avoid the local minima problem, thereby exhibiting improved performance. In future, few other meta-heuristic algorithms like cat swarm optimization, ant lion optimization etc., will be investigated to further improve the classification accuracy.

## Data Availability Statement

The raw data supporting the conclusions of this article will be made available by the authors, without undue reservation.

## Author Contributions

AA: Conceptualization, Methodology, Writing original Draft; PM: Funding Acquistion; MH: Funding Acquisition; SB: Supervision; SA: Resources. All authors contributed to the article and approved the submitted version.

## Conflict of Interest

The authors declare that the research was conducted in the absence of any commercial or financial relationships that could be construed as a potential conflict of interest.

## Publisher’s Note

All claims expressed in this article are solely those of the authors and do not necessarily represent those of their affiliated organizations, or those of the publisher, the editors and the reviewers. Any product that may be evaluated in this article, or claim that may be made by its manufacturer, is not guaranteed or endorsed by the publisher.
